# Coexistence of *tet*(X4), *mcr-1*, and *bla*_NDM-5_ in ST6775 *Escherichia coli* Isolates of Animal Origin in China

**DOI:** 10.1128/spectrum.00196-22

**Published:** 2022-03-21

**Authors:** Xiaoyu Lu, Yufeng Du, Kai Peng, Wenhui Zhang, Jingui Li, Zhiqiang Wang, Ruichao Li

**Affiliations:** a Jiangsu Co-Innovation Center for Prevention and Control of Important Animal Infectious Diseases and Zoonoses, College of Veterinary Medicine, Yangzhou University, Yangzhou, Jiangsu, People’s Republic of China; b Institute of Comparative Medicine, Yangzhou University, Yangzhou, Jiangsu, People’s Republic of China; c College of Pharmacy, China Pharmaceutical University, Nanjing, Jiangsu, People’s Republic of China; d Joint International Research Laboratory of Agriculture and Agri-Product Safety, the Ministry of Education of China, Yangzhou University, Yangzhou, Jiangsu, People’s Republic of China; Forschungszentrum Jülich GmbH

**Keywords:** *Escherichia coli*, ST6775, *tet*(X4), *mcr-1*, *bla*_NDM-5_, coexistence

## Abstract

Emergence of pathogens harboring multiple resistance genes incurs great concerns. Cooccurrence of mobile resistance genes conferring resistance to tigecycline, colistin, and carbapenems in Escherichia coli has not been investigated. This study aimed to characterize three E. coli isolates coharboring *tet*(X4), *mcr-1*, and *bla*_NDM-5_. Isolates coharboring *tet*(X4), *mcr-1*, and *bla*_NDM-5_ were identified and characterized by PCR, Sanger sequencing, antimicrobial susceptibility testing, conjugation assays, Illumina sequencing, nanopore sequencing, and bioinformatic analysis. Three E. coli isolates carrying *tet*(X4), *mcr-1*, and *bla*_NDM-5_ were identified from pigeons in China. They were resistant to almost all antimicrobials except enrofloxacin. *tet*(X4) and *bla*_NDM-5_ could be conjugated into E. coli C600, but *mcr-1* was nontransferable in three isolates. Three isolates belonged to sequence type 6775 (ST6775), and clonal dissemination of isolates carrying *tet*(X4), *mcr-1*, and *bla*_NDM-5_ existed in the pigeon farm. Genetic analysis revealed that *mcr-1* mediated by the Tn*6330* was located on the chromosome, *tet*(X4) was located on the IncFII plasmid, and *bla*_NDM-5_ was located on the IncX3 plasmid. We first characterized the E. coli isolates carrying *tet*(X4), *mcr-1*, and *bla*_NDM-5_ simultaneously. Relevant measures should be taken to decrease the prevalence of pathogens carrying *tet*(X4), *mcr-1*, and *bla*_NDM-5_.

**IMPORTANCE** Tigecycline and colistin are regarded as vital antimicrobials to treat multidrug-resistant (MDR) bacterial infections, such as that caused by carbapenemase-producing *Enterobacteriaceae* (CPE). Cooccurrence of mobile resistance genes conferring resistance to last-resort antimicrobials in E. coli remains unknown. Here, we characterized E. coli strains coharboring *tet*(X4), *mcr-1*, and *bla*_NDM-5_ phenotypically and genetically. Resistance genes *tet*(X4), *mcr-1*, and *bla*_NDM-5_ were located on transposons or plasmids that were mobile genetic elements related to the capture, accumulation, and dissemination of such important resistance genes. The emergence of E. coli isolates carrying *tet*(X4), *mcr-1*, and *bla*_NDM-5_ highlights the importance of monitoring the coexistence of novel mobile resistance genes in different settings with a One Health approach. Risk of transmission of such MDR pathogens from animals to humans should be evaluated comprehensively.

## INTRODUCTION

Antimicrobial resistance is considered a global threat to public health. Carbapenems are critical drugs treating Gram-negative bacterial infections. Accordingly, preventing the spread of carbapenemase-producing *Enterobacteriaceae* (CPE) among different settings continues to present serious challenges (1). New Delhi metallo-β-lactamase (NDM), conferring resistance to almost all β-lactams, is the main carbapenemase, and 40 gene variants, including *bla*_NDM-1_ to *bla*_NDM-40_ (https://www.ncbi.nlm.nih.gov/pathogens/refgene/#NDM), encode NDM enzymes, among which *bla*_NDM-1_ and *bla*_NDM-5_ genes are still the most prevalent variants (2). Colistin and tigecycline are two of the last-resort therapies to treat infections caused by carbapenem-resistant bacterial pathogens (3–5). However, the emergence of plasmid-mediated colistin resistance genes *mcr-1* to *mcr-10* (6, 7) and the plasmid-mediated tigecycline resistance genes *tet*(X3) to *tet*(X15) (8, 9) was reported and attracted public attention. The coexistence of *tet*(X4), *mcr-1*, and *bla*_NDM-5_ will seriously threaten the treatment of carbapenem-resistant bacterial pathogen infections, but no such MDR pathogens are reported currently.

Pigeon is one of the most commonly consumed food animals in Jiangsu, China. Pigeons have close contact to human activities, such as breeders raising pigeons or cleaning pigeon cages. These activities, therefore, may pose potential threats to humans as well as other animals, as pigeons may carry and spread different pathogens, including drug-resistant bacteria. Therefore, the antimicrobial resistance of bacteria isolated from pigeons is worthy of our attention. In this study, we performed an antimicrobial resistance surveillance study of bacteria from pigeons and revealed the molecular characteristics of three ST6775 Escherichia coli isolates coharboring *tet*(X4), *mcr-1*, and *bla*_NDM-5_ from pigeons in China, indicating that the continuous surveillance of such hazardous MDR bacteria should be strengthened to decrease the spread of such MDR bacteria around the world.

## RESULTS

### Isolates’ identification and resistance phenotypes.

Among 100 fecal samples, 3 *tet*(X4)-positive isolates, PT62, PT76, and PT77, were acquired. Strikingly, the *tet*(X4)-positive isolates coharbored *mcr-1* and *bla*_NDM-5_. They were identified as E. coli. Antimicrobial susceptibility testing revealed that all three E. coli isolates conferred resistance to tigecycline, colistin, and meropenem (Table 1). In addition, they exhibited the same resistance spectrum and were phenotypically resistant to almost all other antimicrobials tested, including kanamycin, doxycycline, ampicillin, streptomycin, amoxicillin, florfenicol, oxytetracycline, and tetracycline, but no resistance to enrofloxacin (Table 1). Three isolates were MDR bacteria accordingly.

**TABLE 1:** MICs (mg/L) of three E. coli isolates cocarrying *tet*(X4), *mcr-1*, and *bla*_NDM-5_^*a*^

Strain ID	Antimicrobials:																
	GEN	KAN	DOX	AMP	ENR	CFF	STR	AMX	RIF	CEF	FFC	MEM	IMP	CST	OXY	TET	TIG
PT62	>128	>128	128	>128	≤0.125	>128	64	>128	16	>128	>128	16	16	8	>128	>128	128
PT76	>128	>128	64	>128	≤0.125	>128	32	>128	16	>128	>128	16	16	4	>128	>128	64
PT77	>128	>128	128	>128	≤0.125	>128	64	>128	32	>128	>128	16	16	4	>128	>128	64
ATCC 25922	0.25	2	0.5	4	≤0.125	≤0.125	4	4	4	≤0.125	4	≤0.125	≤0.125	0.25	4	0.5	≤0.125

a GEN, gentamicin; KAN, kanamycin; DOX, doxycycline; AMP, ampicillin; ENR, enrofloxacin; CFF, ceftiofur; STR, streptomycin; AMX, amoxicillin; RIF, rifampicin; CEF, ceftriaxone; FFC, florfenicol; MEM, meropenem; IMP, imipenem; CST, colistin; OXY, oxytetracycline; TET, tetracycline; TIG, tigecycline.

### Transferability of *tet*(X4), *mcr-1*, and *bla*_NDM-5_ genes.

To investigate the transmissibility of *tet*(X4), *mcr-1*, and *bla*_NDM-5_ genes, we subjected three isolates to conjugation experiments with E. coli C600. Results indicated that resistance genes *tet*(X4) and *bla*_NDM-5_ in three isolates, with their corresponding resistance phenotypes for tigecycline and meropenem, were successfully transferred to E. coli C600, suggesting that *tet*(X4) and *bla*_NDM-5_ genes were located on conjugative plasmids or other mobilizable genetic elements in three isolates. The conjugation frequencies of *tet*(X4)-bearing genetic structures were high and ranged from (1.07 ± 0.1) × 10^−1^ to (1.63 ± 0.3) × 10^−1^ transconjugants per recipient. The transfer of *bla*_NDM-5_-harboring genetic structures was at frequencies of (3.95 ± 0.4) × 10^−3^ to (1.10 ± 0.2) × 10^−2^ cells per recipient. Cotransfer of *tet*(X4) and *bla*_NDM-5_ was at frequencies of (2.07 ± 0.1) × 10^−3^ to (3.33 ± 0.3) × 10^−3^ cells per recipient. However, *mcr-1* in three isolates were nontransferable. This indicated that horizontal dissemination of *tet*(X4) and *bla*_NDM-5_ by conjugative plasmids or other mobilizable genetic elements existed in the pigeon farm.

### The clonal relationship analysis based on whole-genome sequencing.

**FIG 1:**
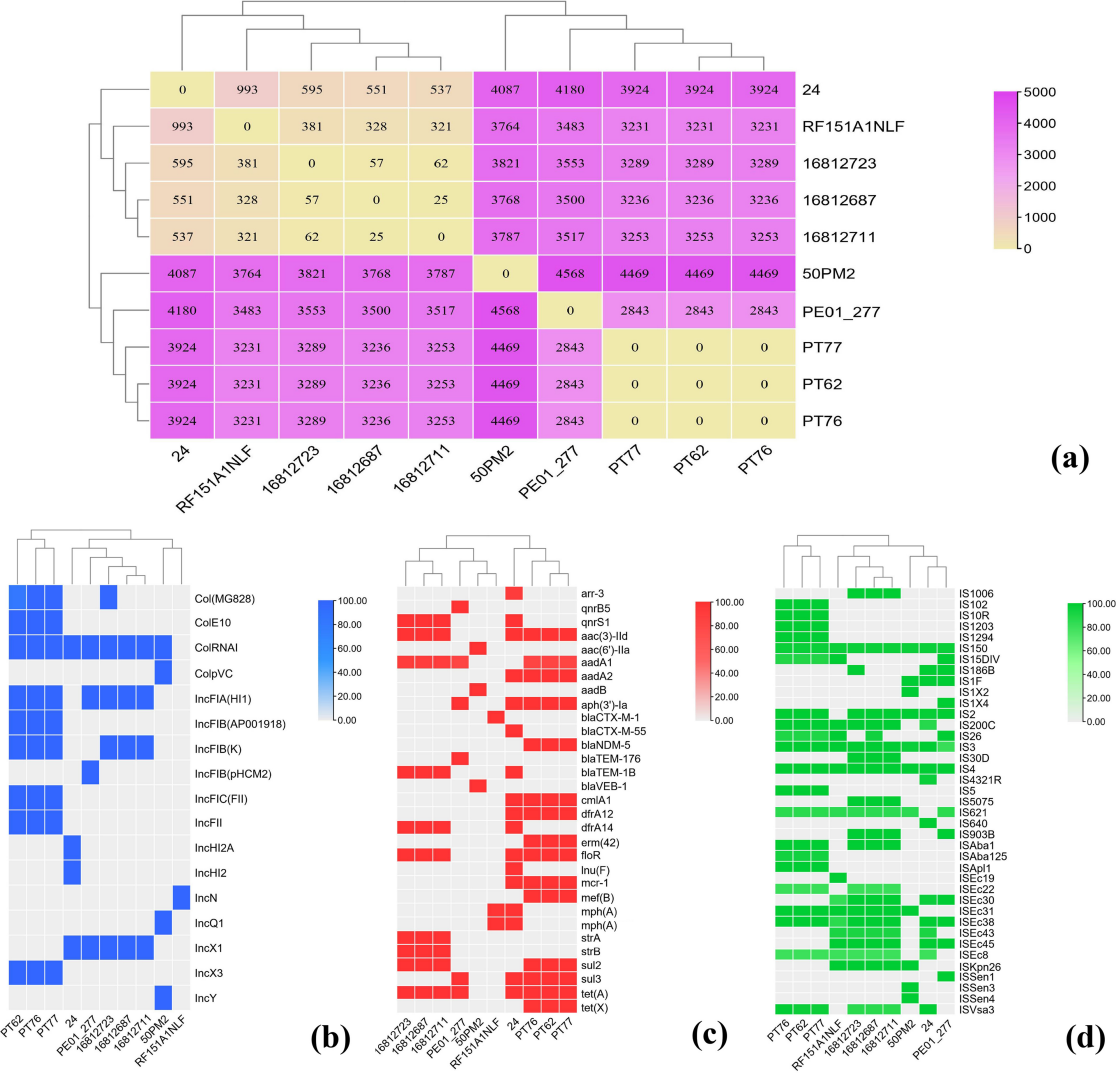
Comprehensive information of the assembled draft genomes from 10 ST6775 E. coli isolates. (a) The number of SNP differences among 10 ST6775 E. coli isolates, including three isolates in the study and seven isolates from the NCBI SRA database. (b) The distribution of plasmid replicons in 10 ST6775 E. coli isolates. The blue rectangles indicate the presence of plasmid replicons. (c) The distribution of antimicrobial resistance genes in 10 ST6775 E. coli isolates. The red rectangles indicate the presence of antimicrobial resistance genes. (d) The distribution of insert sequences in 10 ST6775 E. coli isolates. The green rectangles indicate the presence of insert sequences. Legend labels indicates the similarity (%).

Three isolates were characterized by whole-genome sequencing with the Illumina HiSeq 2500 platform to generate 2 × 150 bp paired-end read data. Multilocus sequence type (MLST) analysis revealed that three isolates positive for *tet*(X4), *mcr-1*, and *bla*_NDM-5_ belonged to ST6775, which was an infrequent ST type. Analysis of the assembled draft genomes from three isolates coharboring *tet*(X4), *mcr-1*, and *bla*_NDM-5_ with seven other ST6775 E. coli isolates from the NCBI SRA database provided comprehensive information. We determined the clonal relationship of 10 ST6775 E. coli isolates based on their single-nucleotide polymorphisms (SNPs) of the core genome. The number of differences in SNPs was 0 between PT62, PT76, and PT77 (Fig. 1a). However, the number of SNP differences between the three isolates carrying *tet*(X4), *mcr-1*, and *bla*_NDM-5_ and the other seven ST6775 E. coli isolates from the NCBI database ranged from 2,843 to 4,469 (Fig. 1a). In addition, all three isolates, PT62, PT76, and PT77, belonged to phylogroup B1 and contained four virulence genes, *gad*, *neuC*, *terC*, and *traT*. They also contained identical plasmid replicons (Fig. 1b), antimicrobial resistance genes (Fig. 1c), and insertion sequences (Fig. 1d), which suggests that clonal dissemination of isolates carrying *tet*(X4), *mcr-1*, and *bla*_NDM-5_ existed in the pigeon farm. Apart from *tet*(X4), *mcr-1*, and *bla*_NDM-5_, 13 resistance genes were identified in each of the three isolates, PT62, PT76, and PT77, including *aadA1*, *aadA2*, *aph(3′)-Ia*, *aac(3)-IId*, *floR*, *cmlA1*, *mdf*(A), *tet*(A), *sul2*, *sul3*, *dfrA12*, *erm*(42), and *mef*(B) (Fig. 1c). These genes confer resistance to diverse antimicrobials, such as aminoglycosides, phenicol, macrolides, tetracyclines, sulfonamides, and lincosamides. Ten ST6775 E. coli isolates were MDR isolates carrying multiple antimicrobial resistance genes, and each isolate carried at least one β-lactamase gene (Fig. 1c). Isolate 24 from the NCBI database also carried the *mcr-1* gene (Fig. 1c). Nine plasmid replicons, Col(MG828), ColE10, ColRNAI, IncFIA(HI1), IncFIB(AP001918), IncFIB(K), IncFIC(FII), IncFII, and IncX3, were found in each of the three isolates PT62, PT76, and PT77 (Fig. 1b). Importantly, we noticed that many other types of plasmids can exist in other ST6775 E. coli from the NCBI database, such as IncHI2 type plasmids where *mcr-1* is often located (10) and IncQ1 type plasmids where *tet*(X4) is often located (11). In addition to PT62, PT76, and PT77, four isolates from the NCBI database also carry IS*CR2* or ΔIS*CR2* (IS*Vsa3*), which provides hot spots for the transposition of *tet*(X4) (Fig. 1d) (8). Relevant measures should be taken to decrease the prevalence of ST6775 E. coli carrying *tet*(X4), *mcr-1*, and *bla*_NDM-5_.

### The sequence features of QitanTech sequencing.

To learn the genetic contexts of *tet*(X4), *mcr-1*, and *bla*_NDM-5_, we carried out the representative isolate PT62 with QitanTech nanopore long-read sequencing to obtain complete genome sequences. A total of 109,949 reads with 748.8 Mb were obtained through the QitanTech nanopore long-read sequencing technology. The mean read length and mean read Phred quality of the total sequencing data were 6,810.5 bp and 8.1, respectively. The median read length and median read Phred quality of the total sequencing data were 3,139 bp and 8.2, respectively. The read length, *N*_50_, of the total QitanTech sequencing data was 15,338 bp. The longest read length was 114,033 bp. The mean basecall quality scores of the top five longest reads (reads were more than 99 kb) were greater than 8.0. Then, the QitanTech long-read sequencing data and Illumina short-read data of isolate PT62 were used to perform *de novo* assembly with the hybrid strategy to obtain the completed chromosome and plasmids. Bioinformatic analysis revealed that isolate PT62 harbored a chromosome and seven plasmids comprising pPT62-tetX-108kb, pPT62-106kb, pPT62-NDM-47kb, pPT62-37kb, pPT62-9kb, pPT62-3kb, and pPT62-1kb (Table 2).

**TABLE 2:** Basic information of the chromosome and plasmids in E. coli PT62

Component	Size (bp)	Accession no.	Replicon type(s)	Resistance gene(s)
PT62-chromosome	4,578,540	CP090448		*mcr-1*, *mdf*(A)
pPT62-tetX-108kb	108,576	CP090449	IncFII	*tet*(X4), *erm*(42), *sul2*, *aac(3)-IId*, *floR*
pPT62-106kb	106,761	CP090450	IncFIB(AP001918), IncFIC(FII)	*aph(3′)-Ia*, *sul3*, *mef*(B)
pPT62-NDM-47kb	47,849	CP090451	IncX3	*bla*_NDM-5_
pPT62-37kb	37,779	CP090452	IncFIA(HI1), IncFIB(K)	*aadA2*, *aadA1*, *cmlA1*, *dfrA12*, *sul3*, *tet*(A)
pPT62-9kb	9,533	CP090453	ColE10	None
pPT62-3kb	2,994	CP090454	ColRNAI	None
pPT62-1kb	1,506	CP090455	Col(MG828)	None

### Genetic environment analysis of *mcr-1*.

**FIG 2:**
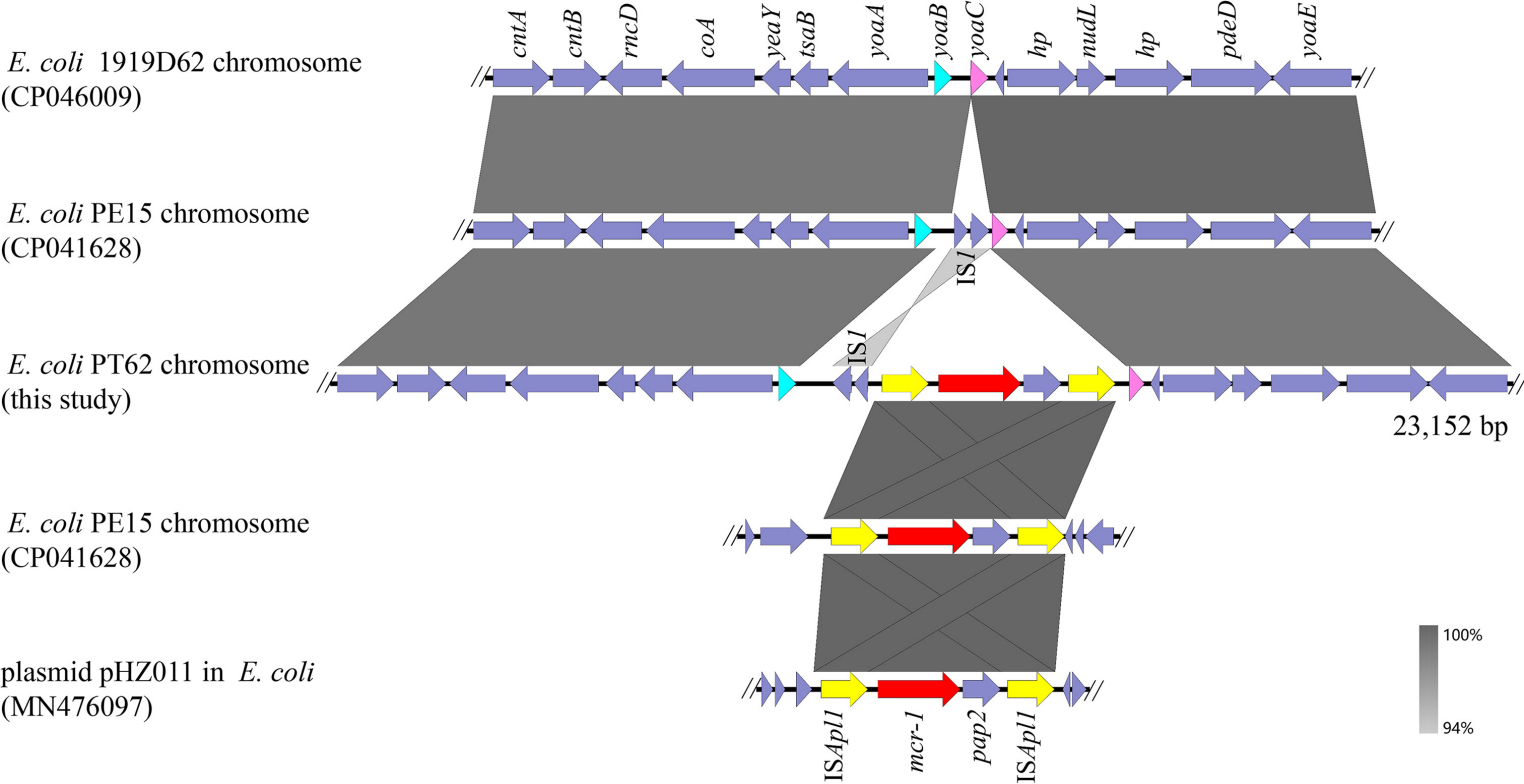
Genetic environment of *mcr-1* in the chromosome. Linear alignment of the selected chromosomal segment (23,152 bp) containing Tn*6330* with other homologous sequences available in the NCBI database.

The *mcr-1* gene in E. coli PT62 was located on the chromosome and mediated by the Tn*6330* (IS*Apl1*-*mcr-1*-*pap2*-IS*Apl1*) mobile element. A chromosomal segment (23,152 bp in length) containing Tn*6330* was selected to perform online BLASTn analysis, which indicated that the segment exhibited 99% identity at 77% coverage with E. coli 1919D62 chromosome (CP046009) without Tn*6330* and 99% identity at 75% coverage against E. coli PE15 chromosome (CP041628) with Tn*6330* appearing in another position of PE15 chromosome (Fig. 2). In addition, the Tn*6330* could also exist on the plasmids (Fig. 2). It has been reported that Tn*6330* could appear on the plasmid and the chromosome in one isolate simultaneously (12). Tn*6330* was believed to play an important role in facilitating the transmission of *mcr-1* (12, 13), therefore expanding the host range. The E. coli PT62 may acquire *mcr-1* by inserting Tn*6330* and IS*1* between genes *yoaB* and *yoaC* (Fig. 2).

### Genetic context analysis of *tet*(X4).

**FIG 3:**
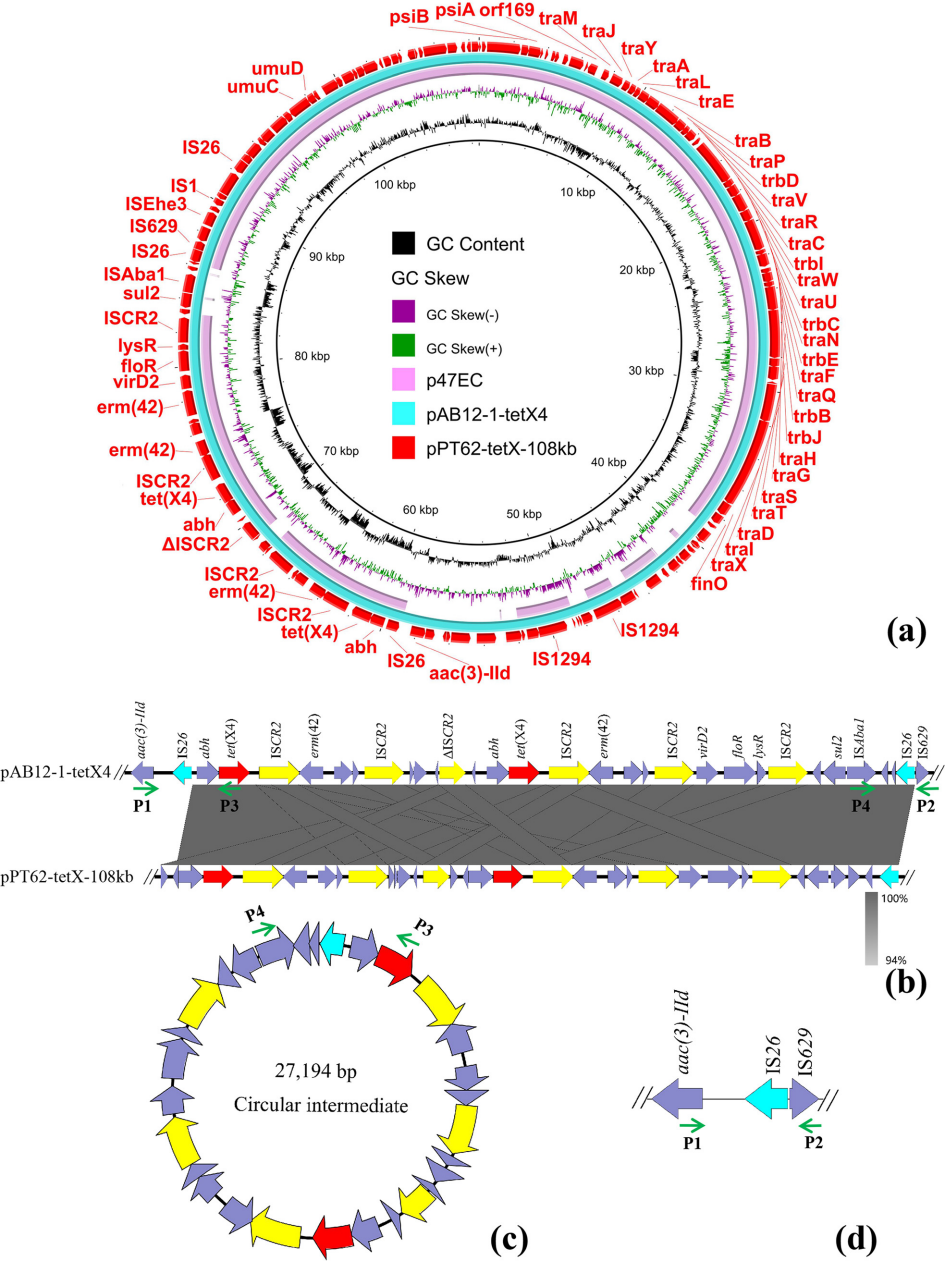
Genetic context of *tet*(X4) in the IncFII plasmid. (a) Circular comparison between the *tet*(X)-bearing IncFII plasmid pPT62-tetX-108kb in the study and other homologous plasmids pAB12-1-tetX4 and p47EC available in the NCBI database. The outermost circles indicate the plasmid pPT62-tetX-108kb with genes annotated. (b) Linear alignment of *tet*(X4)-bearing structure on plasmid pPT62-tetX-108kb with *tet*(X4)-carrying structure on plasmid pAB12-1-tetX4 available in the NCBI database. Green arrows indicate the directions of the primers used to screen the formation of the circular intermediate, and names of primers are marked next to the arrows. (c) The circular intermediate mediated by IS*26*. Green arrows indicate the directions of the primers. (d) A copy of IS*26* left after the circular intermediate fell off. Green arrows indicate the directions of the primers.

The plasmid pPT62-tetX-108kb was a *tet*(X4)-bearing IncFII plasmid with an overall 99% nucleotide identity and 100% query coverage to the sequence of plasmid pAB12-1-tetX4 (MZ054177) in E. coli AB12-1. The plasmid pPT62-tetX-108kb also showed 99% identity (85% coverage) to the sequence of plasmid p47EC (MK134376) in E. coli 47EC (Fig. 3a). In addition, two repeats of *tet*(X4) were found in pPT62-tetX-108kb (Fig. 3a). Various genetic contexts of *tet*(X4) have been reported previously, and all structures were related to IS*CR2* (14). The *tet*(X4)-harboring genetic context in pPT62-tetX-108kb was abundant with insertion sequences, and its structure was IS*26*-*abh*-*tet*(X4)-IS*CR2*-*erm*(42)-*orf*-*hp*-IS*CR2*-*hp*-ΔIS*CR2*-*hp*-*abh*-*tet*(X4)-IS*CR2*-*erm*(42)-*orf*-*hp*-IS*CR2*-*virD2*-*floR*-*lysR*-IS*CR2*-*orf*-*sul2*-IS*Aba1*-*orf*-*hp*-IS*26* (27,917 bp) (Fig. 3b), which was similar to the genetic structure surrounding *tet*(X4) found in plasmid pAB12-1-tetX4, but only one copy of IS*26* was found on the structure of plasmid pAB12-1-tetX4 (Fig. 3b). He et al. reported that the formation of *tet*(X4)-carrying circular intermediate is mediated by IS*CR2*, detected through inverse PCR assays (8). In this study, we designed primers P1, P2, P3, and P4 to verify the formation of *tet*(X4)-bearing circular intermediate caused by IS*26*. We proved that the *tet*(X4)-bearing structure could exist on the plasmid pPT62-tetX-108kb (Fig. 3b) or generate the *tet*(X4)-bearing circular intermediate mediated by IS*26* (Fig. 3c) and leave one copy of IS*26* after the circular intermediate fell off (Fig. 3d). Therefore, the structure flanked by IS*26* is likely to be responsible for the transfer of *tet*(X4) among different plasmids or isolates. The plasmid pPT62-tetX-108kb was an IncFII type MDR plasmid carrying genes *tet*(X4), *erm*(42), *sul2*, *aac(3)-IId*, and *floR* (Table 2). It has been reported that *tet*(X4)-harboring IncFII plasmids with high transfer frequency could coexist with *mcr-1*-bearing IncI2 or IncHI2 plasmids in one isolate (15, 16), implying a potential threat to public health from *tet*(X4)-bearing IncFII plasmids, and the horizontal spread of *tet*(X4)-bearing IncFII plasmids should arouse great attention.

### Genetic environment analysis of *bla*_NDM-5_.

**FIG 4:**
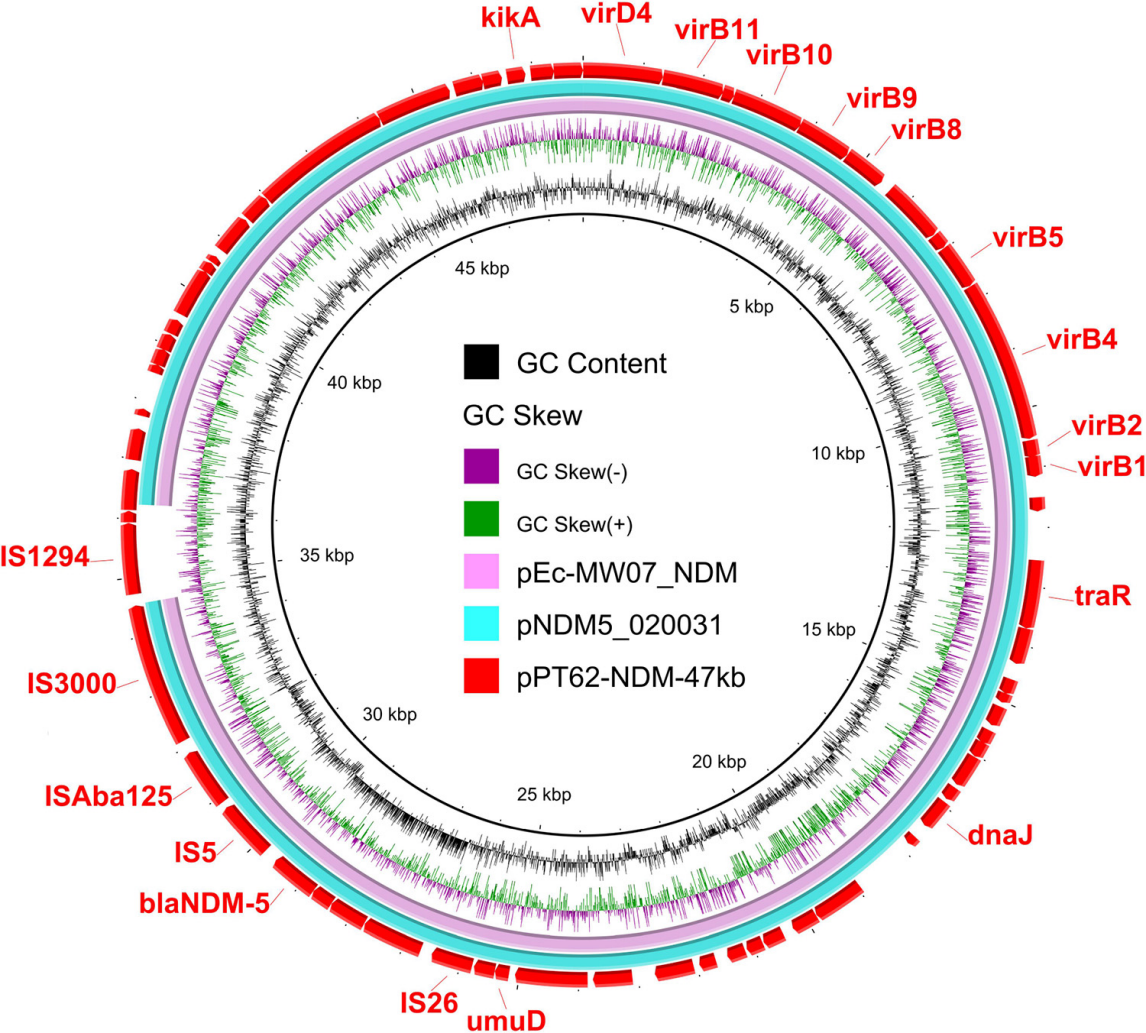
Genetic environment of *bla*_NDM-5_ in the IncX3 plasmid. Circular comparison of three *bla*_NDM-5_-bearing plasmids. The *bla*_NDM-5_-harboring IncX3 plasmid pPT62-NDM-47kb in this study was compared with two plasmids pEc-MW07_NDM and pNDM5_020031 in the NCBI database. The outermost circles indicate the plasmid pPT62-NDM-47kb with genes annotated.

The *bla*_NDM-5_ gene was located on the IncX3 plasmid pPT62-NDM-47kb. The BLASTn analysis indicated that pPT62-NDM-47kb exhibited 100% identity at 96% coverage with plasmid pEc-MW07_NDM (LC545851) from E. coli Ec-MW07 and plasmid pNDM5_020031 (CP033399) from E. coli WCHEC020031 (Fig. 4). No other antimicrobial resistance genes were identified on the plasmid. The *bla*_NDM-5_-bearing genetic structure in pPT62-NDM-47kb was IS*26*-*orf*-*orf*-*orf*-*bla*_NDM-5_-IS*5*-IS*Aba125*-IS*3000*-IS*1294* (Fig. 4). IncX3 type plasmid was an important vector of *bla*_NDM-5_ and has been reported to appear frequently in various sources (17, 18). The similar IncX3 plasmids found in pigeons further highlight the importance of the epidemic IncX3 plasmid in the spread of the *bla*_NDM-5_ gene around the ecosystem. The *bla*_NDM-5_-bearing IncX3 plasmid was frequently found to coexist with *mcr-1*-bearing plasmids in single isolates (19, 20). Recently, the *bla*_NDM-5_-bearing IncX3 plasmid was reported to coexist with *tet*(X4)-harboring plasmid in one isolate (21). There was even one strain in which the *bla*_NDM-4_, *tet*(X4) and *tmexCD3-toprJ3* genes were in a single IncC-IncX3 hybrid plasmid (22). The emergence of isolates’ resistance to carbapenem and tigecycline makes the clinical treatment of carbapenem-resistant pathogens challenging.

## DISCUSSION

The emergence of E. coli isolates conferring resistance to carbapenem and tigecycline from pigeons in this study is alarming because carbapenems and tigecycline are not licensed for use in animals worldwide; however, colistin may have been used in feedstuff as the commercial feed additive before May 2017. Colistin and tigecycline are two of the last-resort therapies to treat infections caused by carbapenem-resistant bacterial pathogens. The presence of ST6775 E. coli isolates carrying tigecycline resistance gene *tet*(X4), colistin resistance gene *mcr-1*, and carbapenem resistance gene *bla*_NDM-5_ from pigeons posed a great public health concern, as therapeutic choices in such cases are very limited. In fact, ST6775 is an infrequent sequence type. It is worth thinking about whether the accumulation of *tet*(X4), *mcr-1*, and *bla*_NDM-5_ in an ST6775 E. coli isolate is related to certain molecular mechanisms or just a coincidence, which warrants further investigations. It is reported that ST410 E. coli isolates could carry *bla*_NDM-5_-bearing IncX3 plasmids (23) or *tet*(X4)-bearing IncFII plasmids (15). Also, chromosomal location of *mcr-1* was found in ST410 E. coli isolate (24). The ST48 E. coli is frequently found to carry the *bla*_NDM-5_-bearing IncX3 plasmid (17) or the *mcr-1*-harboring IncX4 plasmid (25). The coexistence of *tet*(X4)-harboring IncFII plasmid and *mcr-1*-bearing IncHI2 plasmid in an ST48 or ST6751 E. coli isolate has even been reported (16). The *bla*_NDM-5_-bearing IncX3 plasmids were widely detected in ST6751 E. coli isolates (26). Isolates with similar situations are too numerous to mention one by one. It is possible that these ST-type isolates are potential targets for the convergence of three genes *tet*(X4), *mcr-1*, and *bla*_NDM-5_. Preventing the prevalence of isolates with these three genes coexisting is one of our important tasks to curb the development of antimicrobial resistance.

There have been many studies on plasmid fusion mediated by insert sequences, such as IS*26* (14) and IS*CR2* (27). In the study, *tet*(X4)-bearing structure flanking two copies of IS*26* could generate the circular intermediate mediated by IS*26*, suggesting that the structure flanked by IS*26* is likely to be responsible for the transfer of *tet*(X4) among different plasmids or isolates. The *tet*(X4)-harboring genetic context was abundant with IS*CR2* and ΔIS*CR2*; the formation of *tet*(X4)-carrying minicircles mediated by IS*CR2* is also possible (8). Therefore, *tet*(X4)-bearing structure may be able to form recombinant plasmids with other plasmids carrying IS*CR2* or IS*26*, which frequently appear on many MDR plasmids, such as *bla*_NDM-5_-bearing IncX3 plasmids or *mcr-1*-harboring IncHI2 plasmids. The cocarriage of *tet*(X6) and *mcr-1* by a single IncHI2 plasmid (28), and the cocarriage of *bla*_NDM-4_, *tet*(X4), and *tmexCD3-toprJ3* genes by a single IncC-IncX3 hybrid plasmid, have been found previously (22). Previously, we also proved that a single hybrid plasmid bearing *mcr-1* and *tet*(X4) may appear under the mediation of insertion sequences (29). With the emergence of isolates carrying *tet*(X4), *mcr-1*, and *bla*_NDM-5_, the factors affecting plasmid recombination need to be further studied to prevent the emergence and evolution of the plasmids cocarrying *tet*(X4), *mcr-1*, and *bla*_NDM-5_.

**Conclusions.** In conclusion, to the best of our knowledge, this is the first report of the coexistence of *tet*(X4), *mcr-1*, and *bla*_NDM-5_ in ST6775 E. coli isolates of animal origin in China. Alarmingly, *tet*(X4), *mcr-1*, and *bla*_NDM-5_ were located on transposons or plasmids that were mobile genetic elements related to the capture, accumulation, and dissemination of resistance genes. This may lead to increasing prevalence of isolates coharboring *tet*(X4), *mcr-1*, and *bla*_NDM-5_ in the future. Such MDR isolates pose a significant challenge to public health; thus, comprehensive surveillance of such MDR bacteria among different sources as a One Health approach should be utilized. Furthermore, relevant measures should be taken to decrease the prevalence of E. coli carrying *tet*(X4), *mcr-1*, and *bla*_NDM-5_.

## MATERIALS AND METHODS

### Bacterial isolates and identification.

In June 2021, a total of 100 fresh fecal samples were randomly collected from a pigeon farm located in Jiangsu Province, China. Individual fresh fecal samples were collected from 100 different pigeon breeding cages using sterile swabs. Subsequently, each sample was placed in brain heart infusion broth containing tigecycline (2 mg/L) and incubated for 4 h at 37°C with 200 rpm constant shaking. Then, the cultures were plated on MacConkey agar plates supplemented with tigecycline (2 mg/L) and incubated for 14 h at 37°C. One or more colonies with different morphological characteristics from each MacConkey agar plate were purified and subsequently screened for the presence of *tet*(X) by PCR with specific primers and Sanger sequencing as reported previously (8). *mcr* and *bla*_NDM_ were further identified in *tet*(X)-positive isolates using primers described earlier (17, 30). 16S rRNA gene sequencing was performed to confirm bacterial species.

### Antimicrobial susceptibility testing.

The MICs of E. coli isolates coharboring *tet*(X), *mcr*, and *bla*_NDM_ against the antimicrobials (gentamicin, kanamycin, doxycycline, ampicillin, enrofloxacin, ceftiofur, streptomycin, amoxicillin, rifampicin, ceftriaxone, florfenicol, meropenem, imipenem, colistin, oxytetracycline, tetracycline, and tigecycline) were determined using the broth microdilution method in accordance with the Clinical and Laboratory Standards Institute (CLSI) guidelines (31). Results were interpreted according to the CLSI standards and the European Committee on Antimicrobial Susceptibility Testing (EUCAST) breakpoints (http://www.eucast.org/clinical_breakpoints/). E. coli ATCC 25922 served as the quality control strain.

### Conjugation experiments.

To investigate the transferability of *tet*(X), *mcr*, and *bla*_NDM_, we performed conjugation assays as described previously (15). Briefly, each isolate carrying *tet*(X), *mcr*, and *bla*_NDM_ was used as the donor and E. coli C600 (resistant to rifampin) was used as the recipient. Cultures of donors and recipient that reached the 0.5 McFarland culture density were mixed at a ratio of 1:1, respectively. Then, 0.1 mL of the mixed cultures was applied onto LB agar plates. After cultures were kept at 37°C for 12 h, we subsequently collected the bacterial cultures on plates and diluted them in sterile saline. We screened transconjugants carrying *tet*(X), *mcr*, or (and) *bla*_NDM_ on LB agar plates containing corresponding antimicrobials. Antimicrobials were used at the following concentrations: tigecycline, 2 mg/L; colistin, 2 mg/L; meropenem, 2 mg/L; rifampicin, 300 mg/L. The presence of *tet*(X), *mcr*, and *bla*_NDM_ in transconjugants was confirmed by PCR and corresponding resistance phenotyping. Frequencies of conjugation transfer were calculated by the number of transconjugants per recipient.

### Genome DNA sequencing.

The genomic DNA of three isolates were extracted using the FastPure bacteria DNA isolation minikit (Vazyme, China) in accordance with the manufacturer’s recommendations. The concentration and quantity of genome DNA were assessed using the Colibri LB 915 spectrophotometer (Titertek-Berthold, Germany) and gel electrophoresis. The short-read DNA sequencing was performed via Illumina HiSeq 2500 platform, and one representative isolate was further sent out for QitanTech nanopore single-molecule long-read sequencing, which is an emerging nanopore sequencing technology launched by Qitan Technology (QitanTech) in China (32). Short-read and long-read data were acquired with fastq format for further analysis. The quality of sequence data generated by QitanTech nanopore long-read sequencing technology was evaluated using Nanoplot of NanoPack v1.25.0 (33).

### Bioinformatic analysis.

The short-read Illumina raw sequences of three isolates were assembled using SPAdes v3.13.1 (34). Illumina short-read and QitanTech nanopore long-read data were used to perform *de novo* assembly with Unicycler v0.4.8 (35, 36). The Rapid Annotation using Subsystems Technology annotation website server (https://rast.nmpdr.org/rast.cgi) was then used to annotate the assembled genomes (37). The multilocus sequence typing (MLST) was determined using online tool MLST v2.0 (https://cge.cbs.dtu.dk/services/MLST/). Seven available ST6775 E. coli genomes according to the EnteroBase (https://enterobase.warwick.ac.uk/) were acquired from the NCBI SRA database. The plasmid replicons, antimicrobial resistance genes, and virulence genes were analyzed using PlasmidFinder v2.1, ResFinder v4.1, and VirulenceFinder v2.0 (https://cge.cbs.dtu.dk/services/). Insertion sequences were identified using ISfinder v2.0 (https://www-is.biotoul.fr). The assembly genomes were also annotated using Prokka v1.12 (38). A pan-genome analysis was conducted on the samples categorized as ST6775 E. coli isolates using the Roary v3.13.0 (39). SNPs distances were compared within ST6775 E. coli isolates using snp-dists v0.7.0 (https://github.com/tseemann/snp-dists). TBtools v1.098661 was used to visualize the SNPs distances as well as the distributions of antimicrobial resistance genes, plasmid replicons, and insertion sequences (40). Circular comparisons between plasmids were performed using the BRIG v0.95 tool (41). To visualize the genetic comparison features, we used Easyfig v2.2.3 to generate linear comparison figures (42).

### Verification of the circular intermediate formation.

Four primers, P1 (5′-TCTCCTCGTAGGGTGATCGG-3′), P2 (5′-AGCCTTCCCAGCAATCGTC-3′), P3 (5′-TAGTCAGTCCAACGGGTCCA-3′), and P4 (5′-TTCAAATGGCGATTCAGCGT-3′), were designed to determine whether homologous recombination between two copies of IS*26* in the same direction could result in the formation of *tet*(X4)-bearing circular intermediate. Because the complete *tet*(X4)-containing structure is too long to be directly amplified by PCR, primer pairs P1 and P3 (PCR product was 2,753 bp), and P2 and P4 (2,021 bp) were employed to prove that the structure exists on the *tet*(X4)-bearing plasmid. Primer pair P3 and P4 (2,679 bp) was used to confirm the formation of the circular intermediate. Primer pair P1 and P2 (2,095 bp) was employed to prove that only one copy of IS*26* was left after the circular intermediate fell off. The PCR products were further purified and then confirmed by Sanger sequencing using the same primers.

### Nucleotide sequence accession numbers.

The nucleotide sequences of the chromosome and plasmids of E. coli PT62 have been deposited in the NCBI database with GenBank accession numbers CP090448 to CP090455 (Table 2). The draft genomes of E. coli PT76 and PT77 are also available in the NCBI database (BioProject accession number PRJNA780102).
